# Dyedauxiliary Groups, an Emerging Approach in Organic Chemistry. The Case of Arylazo Sulfones

**DOI:** 10.1021/acs.joc.0c01895

**Published:** 2020-09-21

**Authors:** Di Qiu, Chang Lian, Jinshan Mao, Maurizio Fagnoni, Stefano Protti

**Affiliations:** † Tianjin Key Laboratory of Structure and Performance for Functional Molecules, College of Chemistry, Tianjin Normal University, Tianjin 300387, P.R. China; ‡ PhotoGreen Lab, Department of Chemistry, University of Pavia, V. Le Taramelli 12, Pavia 27100, Italy

## Abstract

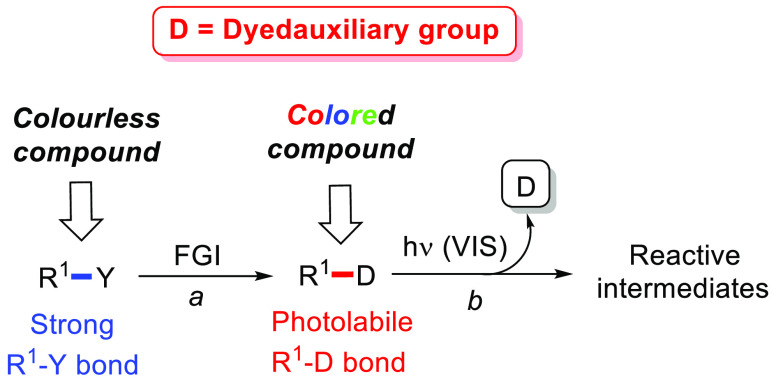

The number of research papers that report photocatalyst-free protocols is currently increasing. Among the different approaches proposed, the conversion of a strong C–X bond of a stable substrate into a photolabile reactive moiety has been recently proposed. In this Synopsis, we introduce the so-dubbed *dyedauxiliary group* strategy by focusing on arylazo sulfones that are bench stable and visible-light responsive derivatives of anilines that have been exploited as precursors of a wide range of intermediates, including carbon-centered radicals as well as aryl cations.

The development of successful synthetic procedures able to satisfy simultaneously the needs for selectivity, efficiency, and sustainability has been considered for a long time as the *holy grail* for every organic chemist. Along with catalysis, photochemistry has always offered a valuable contribution to this target since the light is exclusively responsible for the activation of the substrate. Accordingly, the efficient generation of a reactive intermediate occurs without the intermediacy of either aggressive reactants or harsh conditions.^1^ As a matter of fact, the photon is the greenest reactant that activates the substrate without leaving traces at the end of the process;^2^ unfortunately, most organic compounds are colorless, thus imposing the use of expensive apparatuses and dedicated equipment.^3^ However, the current availability of low energy-demand visible-light sources (e.g., LEDs, compact fluorescent lamps) and “infinitely available” sunlight^4^ has forced the photochemical community to find chemical systems able to absorb such photons. A way to overcome this hurdle is by having recourse to visible-light photocatalysis where a colored compound has the role of absorbing the radiation and promoting the elaboration of colorless compounds.^5^

In the simplest scenario, however, photons should be directly absorbed by one of the colored reactants, thus inducing the chemical event under photocatalyst-free conditions. Though natural and artificial colored compounds are widely present, their direct photochemistry is not of practical interest,^6^ apart from the case of diarylazo compounds, which found sparse application in supramolecular chemistry as photoswitches^7^ and molecular machines,^8^ and the case of α-diketones.^9^

Different approaches to obtain a colored, (photo)reactive moiety in solution have been elaborated. The best known is the formation of an electron donor–acceptor (EDA) complex obtained via the interaction occurring between colorless compounds upon mixing where visible-light irradiation of the resulting chromophore led to the desired products.^10^

Alternatively, a chromophore activation strategy can be adopted.^11^ This involves the use of an additive (e.g., a Brønsted or a Lewis acid) to complex a colorless compound causing a bathochromic shift of the absorption spectrum to the visible region. This reversible complexation induced a spectroscopic change such as the conversion of enone dithianes and dithiolanes **1** into colored thionium ions **2** that underwent a visible-light-induced [2 + 2] photocycloaddition under acid catalysis at a low temperature (Figure 1).^11^

**Figure 1:**
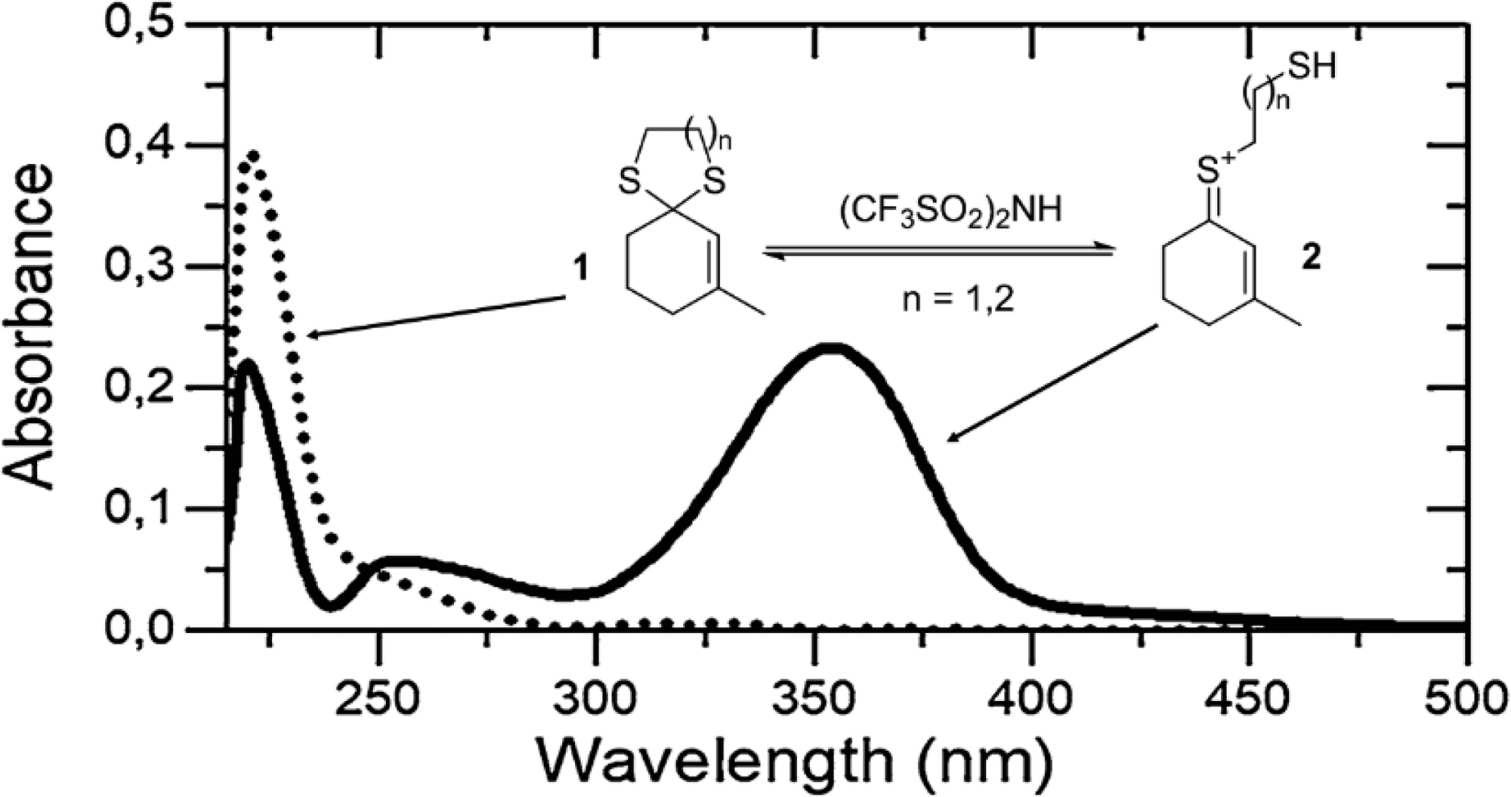
UV/vis absorption spectra of compound **1** in CH_2_Cl_2_ solution without (···) and in the presence (—) of a Brønsted acid (Tf_2_NH). Adapted with permission from ref (11b). Copyright 2018 Springer Nature.

A colored compound may, however, engage a bimolecular reaction (usually via a Single Electron Transfer, SET, process) upon light absorption. Representative cases are the functionalization of colored cyanoarenes (9,10-dicyanoanthracene, DCA, and 2,6,9,10-tetracyanoanthracene, TCA) in the presence of electron donors^12^ or of 4-alkyl-1,4-dihydropyridines in the presence of electron acceptors.^13^

A more intriguing situation is observed when the colored compound can release photochemically reactive intermediates such as radicals without the need for a photocatalyst. In the last five years, we outlined the concept of *dyedauxiliary group* (Figure 2), a moiety able to impart both color and photoreactivity to an organic molecule.

**Figure 2:**
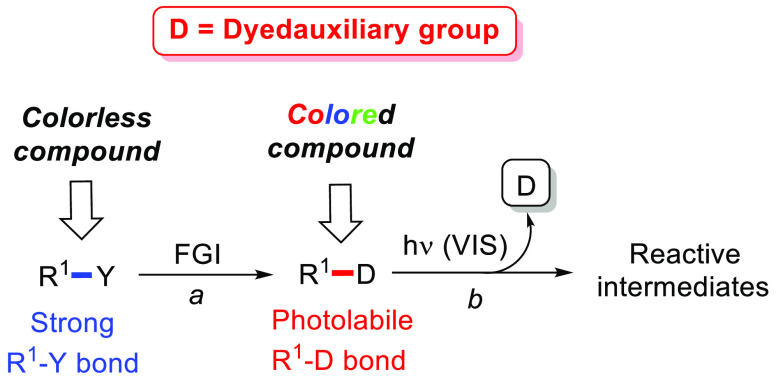
Dyedauxiliary group strategy for the generation of reactive intermediates.

This must exhibit three different properties:

(a) The incorporation of a dyedauxiliary group (D, path a) via functional group interconversion (FGI) in an organic compound (having a strong R^1^–Y bond) makes the organic compound able to absorb in the visible light region.
(b) The resulting R^1^–D bond must be photolabile to generate the desired reactive intermediate.
(c) The mechanism of dyedauxiliary photoremoval should not depend on the nature of the R^1^ group, to ensure a large versatility of the method.

The use of such dyedauxiliary groups is sparsely reported, with Barton esters (Scheme 1a) and acyl xanthates the prototypical examples. In the first case, the strong R^1^–C bond in the starting carboxylic acid is made photolabile by the introduction of a thiohydroxamate chromophore. Photoinduced homolysis of the N–O bond releases a carbonyloxy radical that, after the loss of carbon dioxide, furnished a (substituted) carbon-centered radical.^14^ Acyl xanthates are easily prepared by treatment of an acid chloride with a xanthate salt and exploited as a source of either acyl or alkyl radicals upon visible-light exposition.^15^

**Scheme 1:**
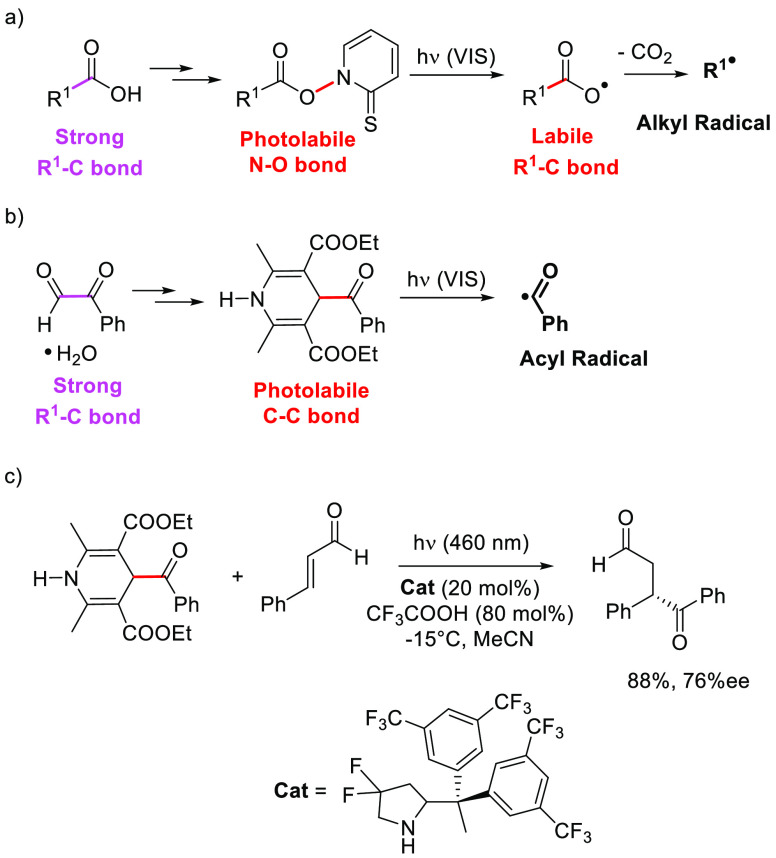
Generation of Chemical Intermediates via Visible-Light-Driven Photolysis of (a) Barton Esters, (b) a 4-Benzoyl-1,4-dihydropyridine, and (c) an Example of the Approach Described in (b)

A more recent example deals with the conversion of a stable colorless glyoxal hydrate into a colored 4-benzoyl-1,4-dihydropyridine having a photolabile C–C bond prone to release an acyl radical upon direct photocleavage (Scheme 1b).^16^ This behavior has been exploited in asymmetric catalytic transformations (Scheme 1c). The conversion of a benzyl bromide into the corresponding 2,3,6,7-tetrakis(tetramethylguanidino)pyridinium salt was likewise reported to promote visible-light-driven benzyl radical dimerization.^17^ Analogously, dithiocarbamate anion was used as a catalyst to transform alkyl halides into colored and photoreactive precursors of several carbon-centered radicals.^18^

One of the most recent examples of dyedauxiliary group is represented by the −N_2_SO_2_R substituent in (hetero)arylazo sulfones **5**. Such thermally stable and colored derivatives can be smoothly prepared from the corresponding anilines **3** (via conversion to diazonium salts followed by coupling with sulfinate salts, Scheme 2, path a)^19^ or via oxidation of *N*-sulfonylaryl hydrazines, in turn generated from aryl hydrazine **4** (path b).^20^ Since the discovery of their photoreactivity, sulfones **5** should be stored under exclusion of light.

**Scheme 2:**
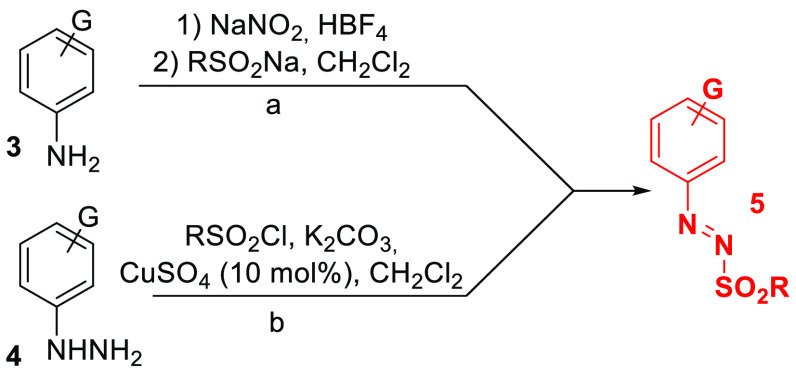
Preparation of Arylazo Sulfones **5** from (a) Anilines and (b) Aryl Hydrazines

Compounds **5** have been investigated in the past, and their decomposition at high temperatures^21^ or in the presence of strong acids^22^ and bases (e.g., CaO or pyridine)^21b,23^ was reported to release aryl cations and aryl radicals. The synthetic potential of arylazo sulfones, however, received only little attention: examples include the preparation of iodoarenes^24^ and their use as electrophiles in the reaction with Grignard reagents,^25^ selenolate and tellurate anions,^26^ as well as dienophiles in [3 + 2] cycloadditions.^27^

Concerning their photophysics, such compounds exhibit two absorption maxima, located in the UV (300–360 nm, ε = 10000–20000 M^–1^ cm^–1^) and in the visible region (400–450 nm, ε = 100–200 M^–1^ cm^–1^, see an example in Figure 3) that have been assigned to a ππ* and an nπ* transition, respectively.^28^

**Figure 3:**
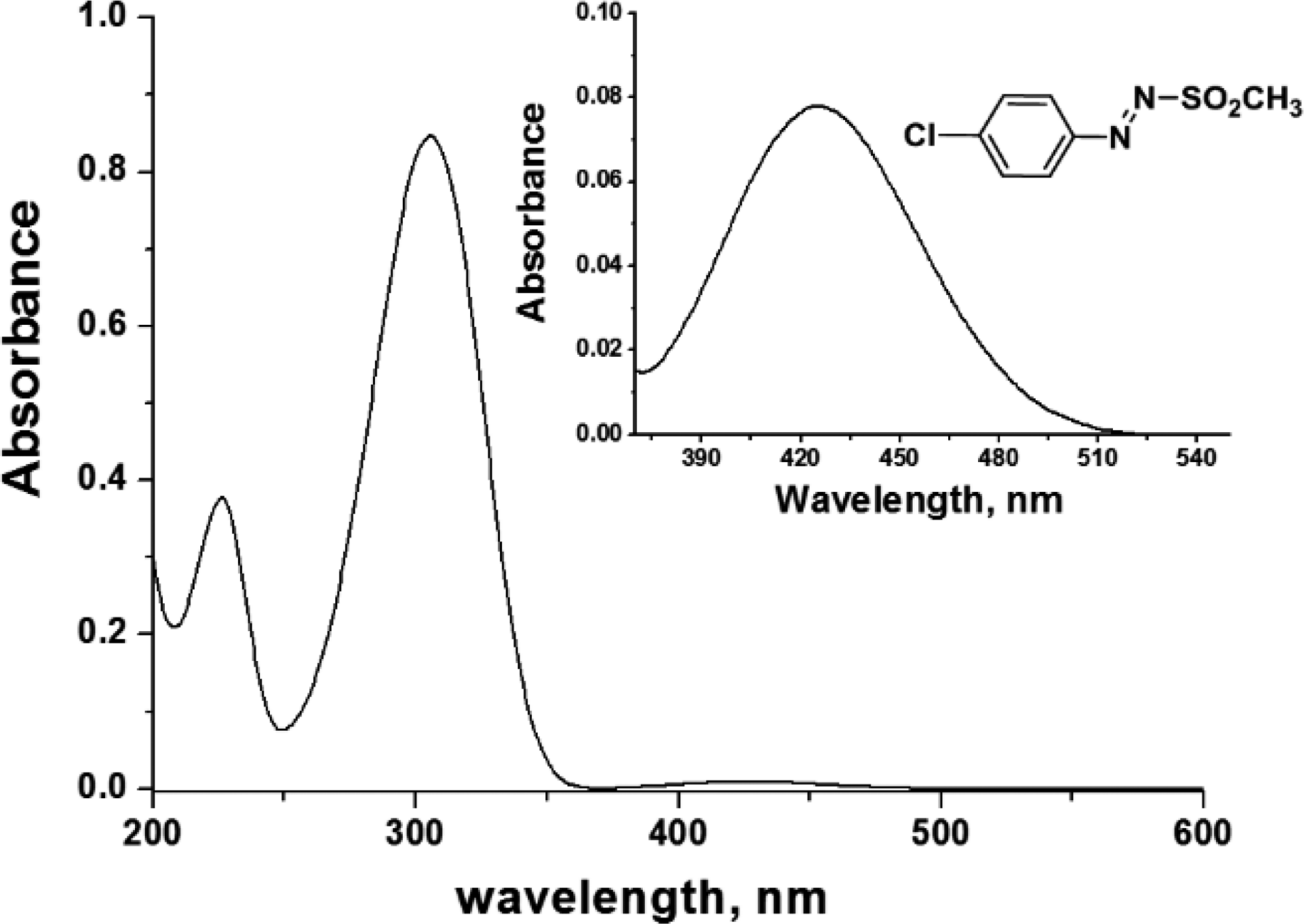
UV absorption spectrum of a 5 × 10^–5^ M solution of 4-chlorophenylazo methylsulfone in acetonitrile. Inset: absorption in the visible region (5 × 10^–4^ M).

The photochemical generation of aryl radicals from arylazo sulfones was suggested in the early 1970s,^22^ but only recently has a detailed investigation been performed.^28^ As a matter of fact, the observed photoreactivity depends on the populated excited state in a wavelength-dependent fashion.^29^ Thus, upon UV irradiation, the generated ^1^ππ* state undergoes intersystem crossing (ISC) to the corresponding triplet (^3^ππ*, Scheme 3 path a), and heterolysis of the N–S bond takes place to release a diazonium salt with the same multiplicity (^3^ArN_2_^+^, path b). The latter, upon dediazoniation (path c), is converted in a triplet phenyl cation (^3^Ar^+^) along with methanesulfinate anion as the counterion.^28^ On the other hand, visible-light exposition populates selectively the ^1^nπ* state of **5** and homolysis of the N–S bond generates, after nitrogen loss from the diazenyl radical Ar–N_2_^•^, an aryl (Ar^•^)/methanesulfonyl (CH_3_SO_2_^•^) radical pair (paths e,f). It is, however, believed that isomerization of the N=N bond from the *trans* to the (less stable) *cis* configuration plays a key role in the cleavage of the N–S bond.^30^ Both aryl cations and aryl radicals are accessible when a polychromatic light (e.g., sunlight) is used as the energy source.^28^

**Scheme 3:**
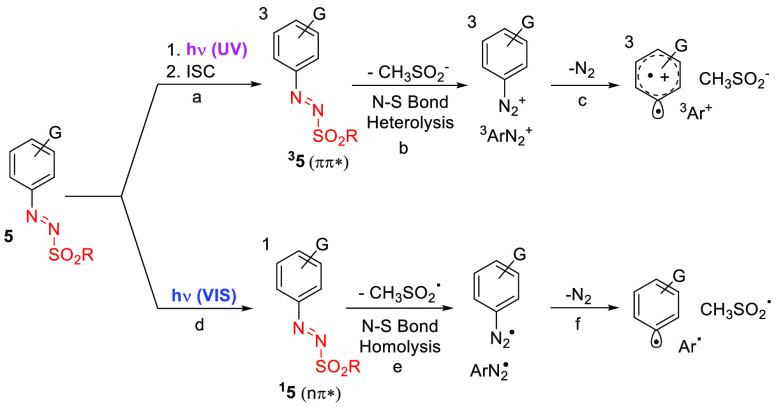
Photochemistry of Arylazo Sulfones **5**

The application of these electrophiles in synthesis has been widely described.^29,31^ In this context, the chance of generating both cations and radicals from arylazo sulfones under mild and (photo)catalyst-free conditions spurred some research groups to consider them as promising substrates in organic chemistry.

## Arylazo Sulfones in Aryl–C Bond Formation

**Scheme 4:**
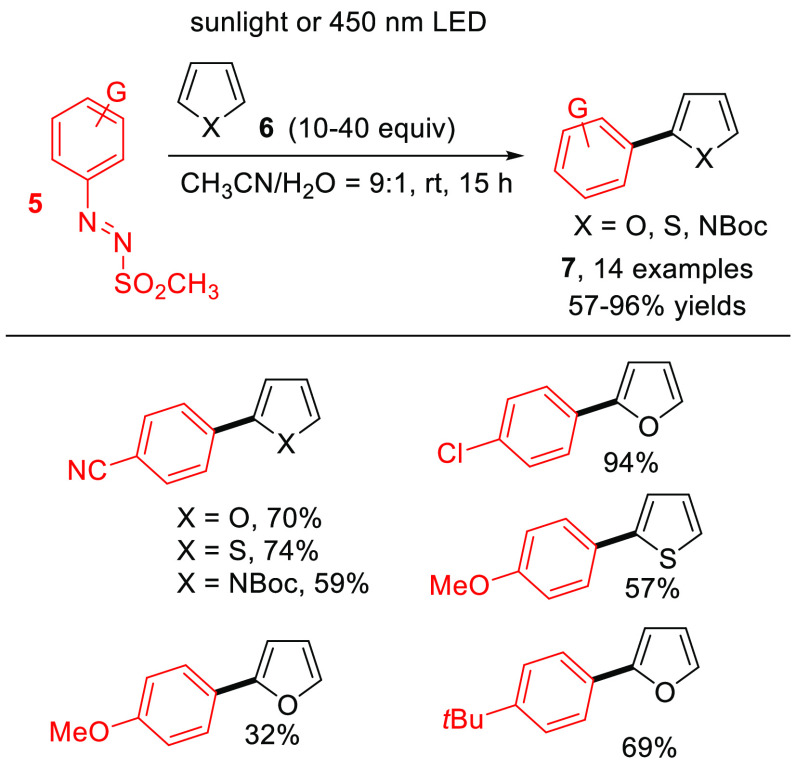
Gomberg–Bachmann Photoarylation via Arylazo Sulfones **5** (General Procedure and Selected Examples)

As notes above, Minato and co-workers previously reported the photolysis of phenylazo *p*-tolyl sulfones^22^ by means of a high-pressure mercury lamp to form the corresponding biaryls by using aromatic media as coupling partners. In 2016, we developed a protocol for the formation of Ar–Ar bonds via both visible and sunlight irradiation of arylazo sulfones in the presence of different heteroaromatics **6** (Scheme 4).^28^ The reaction allowed for the preparation of various heterobiaryls **7** in satisfactory yields without the intermediacy of any (photo)catalyst or additive.

**Scheme 5:**
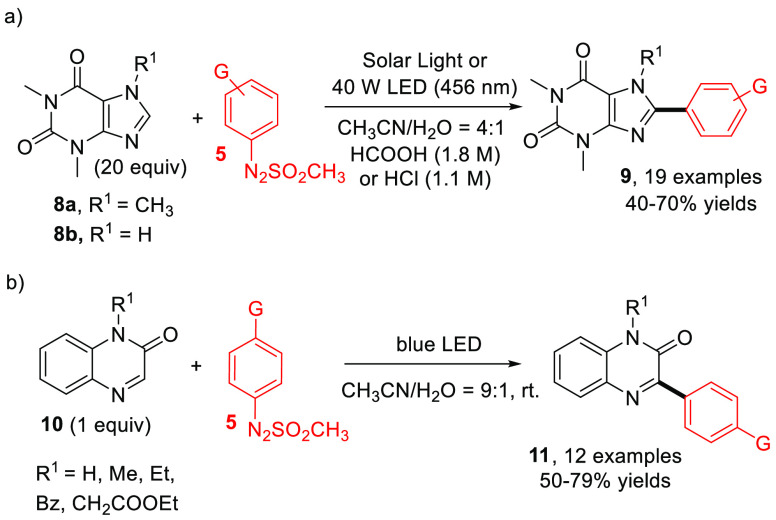
Visible Light Driven Arylation of (a) Xanthines and (b) Quinoxalin-2(1*H*)-ones

The same reactions were also optimized (in three different geographical locations, Germany, Italy, and Brazil) under flow conditions by adopting a solar microcapillary reactor (the so-called “Sunflow” apparatus),^32^ a device that allowed for an efficient conversion of the substrate after only 1 h of exposition to natural sunlight. A similar approach was exploited for the direct C–H arylation of caffeine **8a** and theophylline **8b** in aqueous acidic media.^33^ Indeed, it was demonstrated that the biological performance of a xanthine is significantly improved by the presence of an aryl group at the 8-position (as in compound **9**, Scheme 5a).^34^ The process can be performed successfully also by using a 456 nm Kessil Lamp as the light source. In a similar way, 3-arylquinoxalin-2(1*H*)-ones **11**, a moiety diffused in several enzyme inhibitors and anticancer agents,^35^ has been achieved by using arylazo sulfones as the photoarylating agents.^36^

**Scheme 6:**
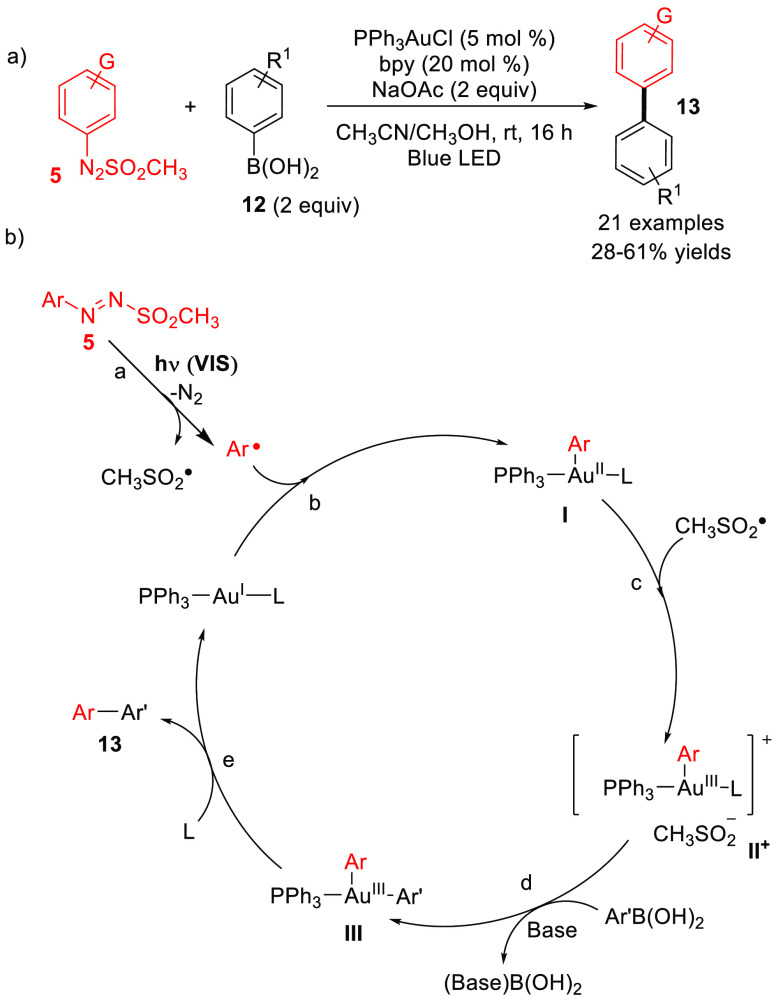
(a) Visible-Light-Driven Gold-Catalyzed Suzuki Synthesis of (Hetero)biaryls. (b) Proposed Mechanism bpy = 2,2′-bipyridine.

In recent decades, the interaction between transition-metal catalysts and carbon-centered radicals was the object of interest for promoting valuable ipso-substitutions in the aromatic ring. In this regard, the dual visible-light/gold-catalyzed Suzuki-type coupling of arylazo sulfones with arylboronic acids (Scheme 6a) gives access to a variety of (hetero)biaryls in moderate to good yields under visible-light-assisted regime and mild conditions.^37^ The reaction mechanism proceeds as illustrated in Scheme 6b. The oxidative addition of Ar^•^ (generated from **5**) onto the Au(I) catalyst generated the Au(II) species **I**, which was further oxidized by the methanesulfonyl radical (CH_3_SO_2_^•^) and afforded the Au(III) adduct **II**^**+**^. Nucleophilic substitution at the Au(III) center by the aryl boronic acid, and the subsequent reductive elimination, resulted in the formation of coupling product **13** while restoring the Au(I)-based catalyst.^37^

**Scheme 7:**
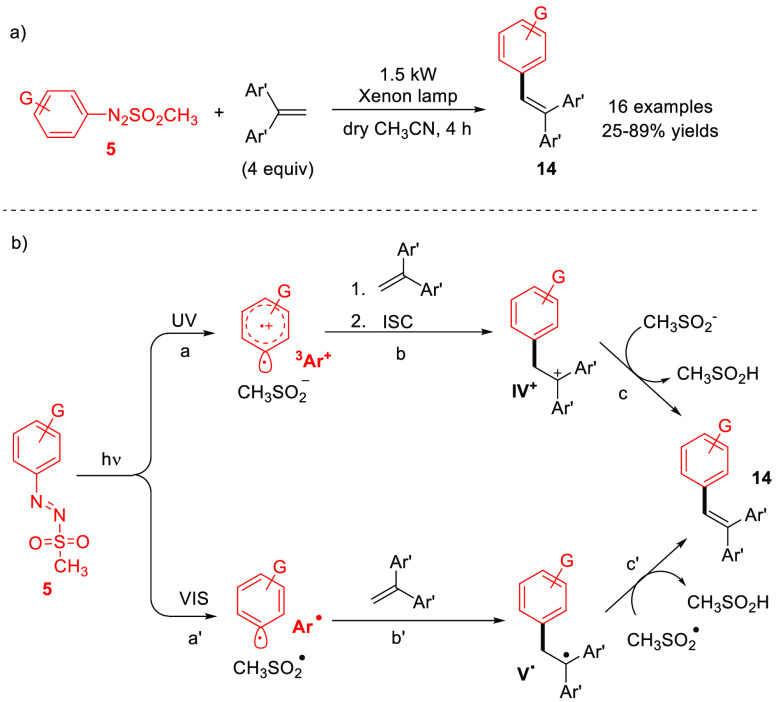
Visible-Light-Driven Synthesis of Triarylethylenes **14** via Metal-Free Heck-Type Coupling between Arylazo Sulfones **5** and 1,1-Diarylethylenes

An alternative approach to forge an Ar–C(sp^2^) bond is via arylation of alkenes to have access to substituted triarylethylenes (TAEs, **14**, Scheme 7).^38^ The reaction proceeds in a solar simulator equipped with a 1500 W xenon lamp (able to simulate the solar emission spectrum) as the photochemical reactor. Noteworthy, the unreacted diphenylethylenes were easily recovered during the purification step. According to the photoreactivity of **5**, both triplet aryl cations (path a) and aryl radicals (path a′) are generated upon sunlight exposition and the two intermediates are efficiently trapped by 1,1-diaryl ethylenes (path b, b′) to form a phenethyl cation (**IV**^**+**^) and a radical (**V**^**•**^), respectively. Deprotonation of **IV**^**+**^ by the methanesulfinate anion (CH_3_SO_2_^–^, path c) and hydrogen atom abstraction from **V**^**•**^ (path c′) operated by CH_3_SO_2_^**•**^ afforded the desired **14** in a convergent fashion.^38^

**Scheme 8:**
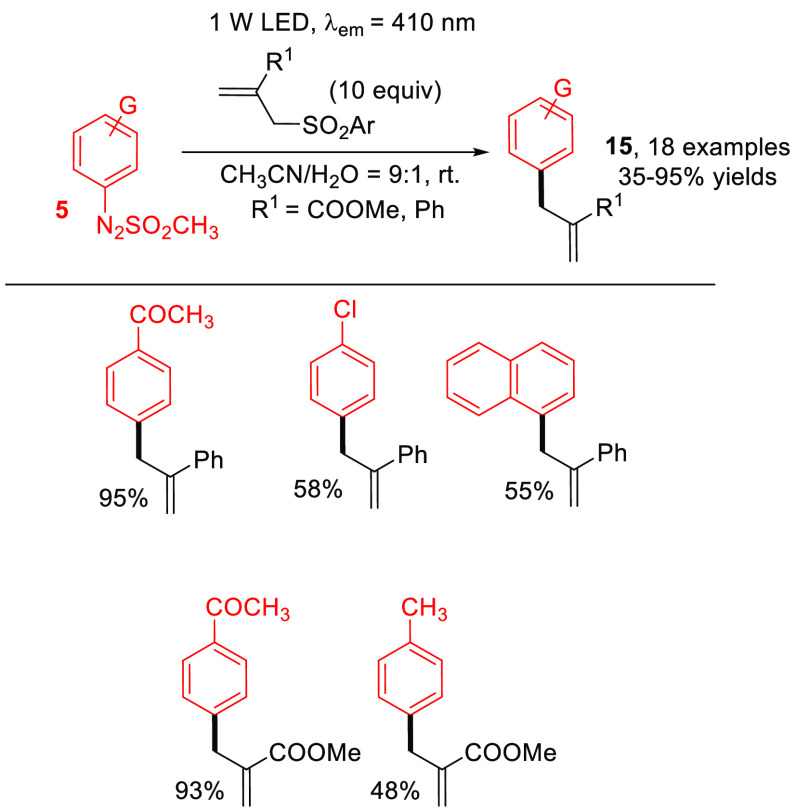
Metal-Free Synthesis of Allyl Arenes **15** (General Procedure and Selected Examples)

The conventional approaches for the allylations of arenes suffered from harsh reaction conditions or contamination of the products by heavy metals.^39^ In this context, arylazo sulfones have been used in the synthesis of allyl arenes **15** starting from α-benzyl styrenes and 2-benzyl acrylates (Scheme 8) as the coupling partners. Again, the purification step allowed for an efficient recovery of the unreacted allylating agent.^40^

**Scheme 9:**
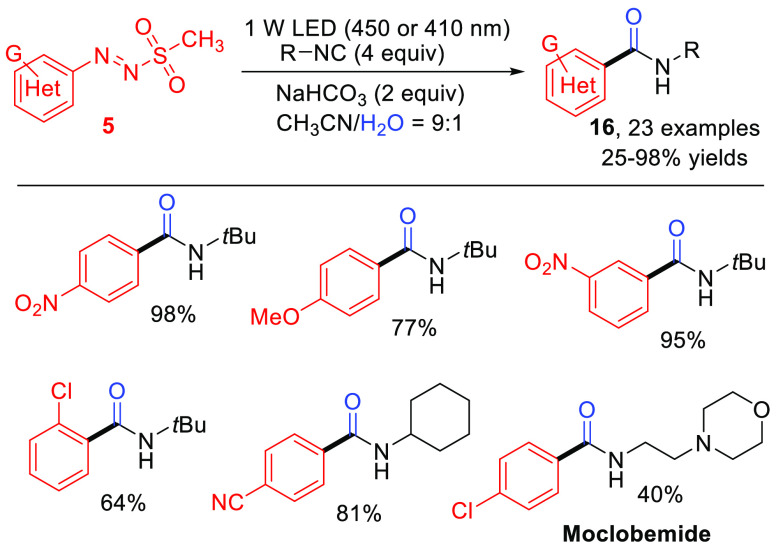
Visible-Light-Driven Route to Aromatic Amides via Radical Arylation of Isonitriles (General Procedure and Selected Examples)

The use of isocyanides for the introduction of an amide group onto an aromatic ring has recently attracted attention.^41^ A visible-light-driven, metal-free synthetic way to aromatic amides **16** (including the antidepressant moclobemide) was achieved via radical arylation of isonitriles using arylazo sulfones as suitable precursors of aryl radicals (Scheme 9) in aqueous acetonitrile.^42^

## Arylazo Sulfones in Aryl–X Bond Formations

**Scheme 10:**
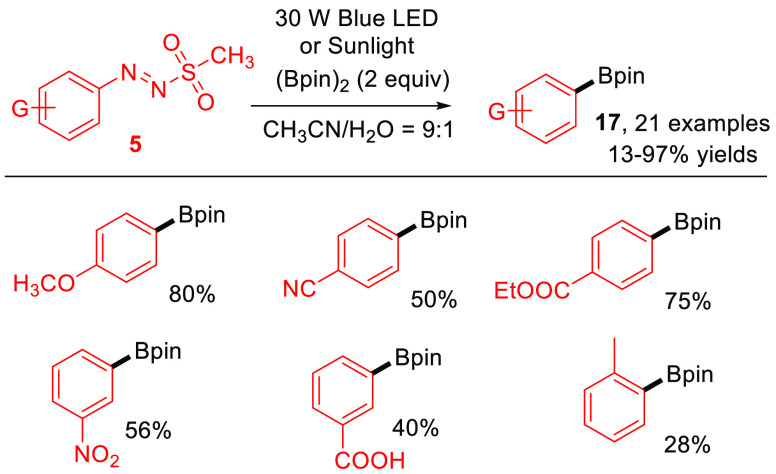
Metal-Free Synthesis of Aryl Boronates **17** (General Procedure and Selected Examples)

Arylazo sulfones also have been used to build aryl–X bonds. Aryl boronic acids and aryl boronates find widespread applications as arylating agents in the Suzuki–Miyaura cross-coupling reactions. Different photochemical procedures for their preparation have been reported,^43,44^ but additives or photosensitizers are mandatory for the success of the process. In 2018, Fang and co-workers proposed a photocatalyst- and additive-free visible-light-induced borylation of arylazo sulfones **5** to afford substituted aryl boronates **17** in high yields by using a diboron reagent as the borylating agent (Scheme 10).^45^ An analogous formation of Ar–B bonds was later reported, having recourse to cyclic diboranes.^46^

**Scheme 11:**
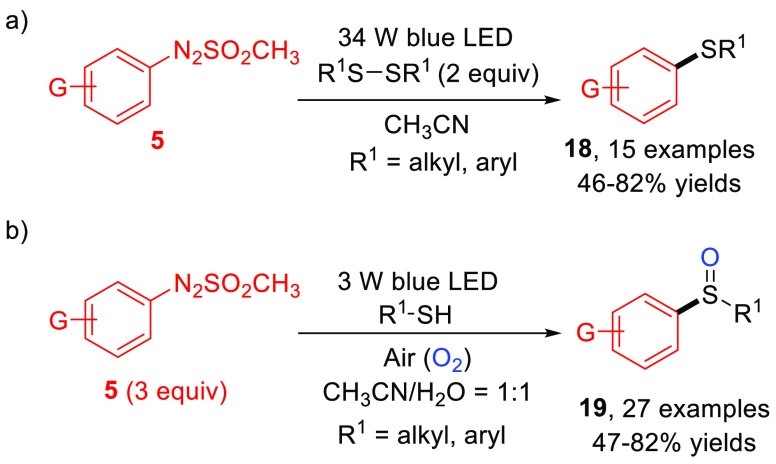
Visible-Light-Promoted Oxidative Coupling of Arylazo Sulfones Leading to Unsymmetrical Sulfoxides

The formation of Ar–S bonds for the synthesis of aryl sulfides **18** starting from dialkyl and diaryl disulfides (Scheme 11a) was also reported.^46^ In 2019, Wei described a catalyst-free visible-light-induced synthetic method for the preparation of a variety of functionalized unsymmetrical sulfoxides **19** via irradiation of **5** in the presence of commercially available thiols in air saturated atmosphere (Scheme 11b).^47^ This strategy displayed several advantages such as high selectivity, mild conditions, and good functional group tolerance.

**Scheme 12:**
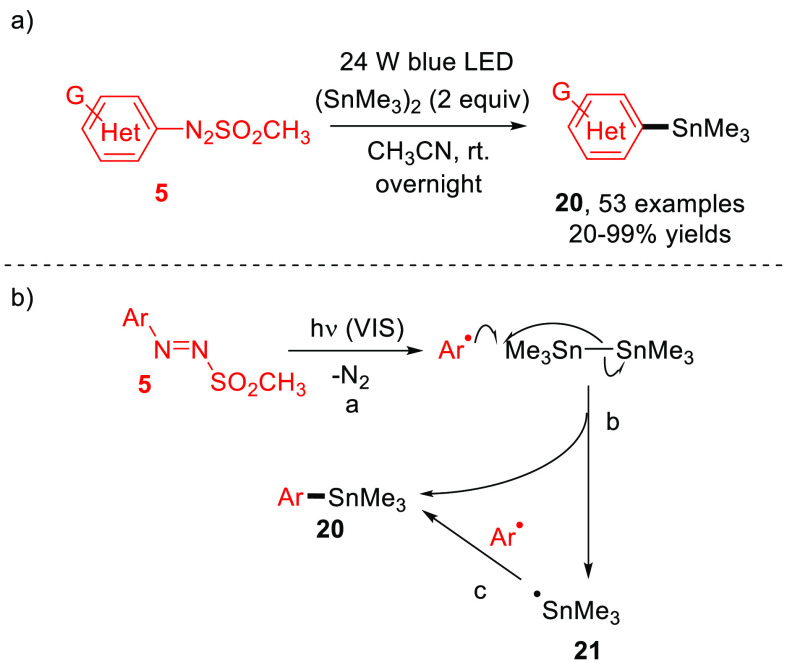
Visible-Light-Driven Synthesis of Aryl Stannanes **20**

Organotin derivatives (especially aryl stannanes) are widely used in organic synthesis.^48^ In 2019, we achieved a visible-light-driven preparation of (hetero)aryl stannanes **20** under both photocatalyst- and metal-free conditions (Scheme 12a).^49^ This mild protocol features high efficiency and extremely wide substrates scope, and the stannylation may be easily scaled to gram-scale amounts. The reaction occurs via the pathway illustrated in Scheme 12b, as demonstrated by mechanistic investigations. Indeed, aryl and heteroaryl radicals generated via blue light excitation of **5** (path a) react with (Me_3_Sn)_2_ to give the desired product **20** along with Me_3_Sn^•^ radical **21** (path b). The direct radical combination of Ar^•^ with Me_3_Sn^•^ is another possible route to reach **20** (path c).^49^

**Scheme 13:**
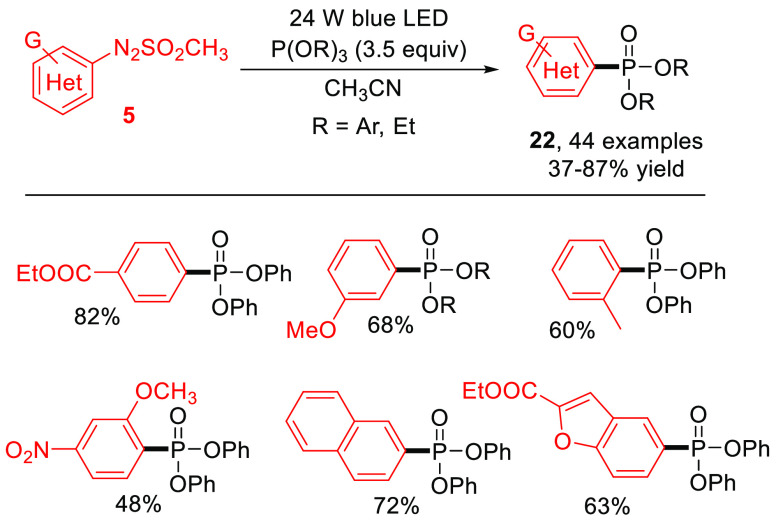
Visible-Light-Driven Phosphonylation of **5** (General Procedure and Selected Examples)

Arylazo sulfones have been adopted for the construction of C–P bonds by employing triaryl (or trialkyl) phosphites as the phosphorus sources.^50^ The reaction gives functionalized (hetero)aryl phosphonates **22** in moderate to good yields (Scheme 13) and exhibits a wide substrates scope, especially for the excellent compatibility to electron-rich arenes and (hetero)aromatics.

**Scheme 14:**
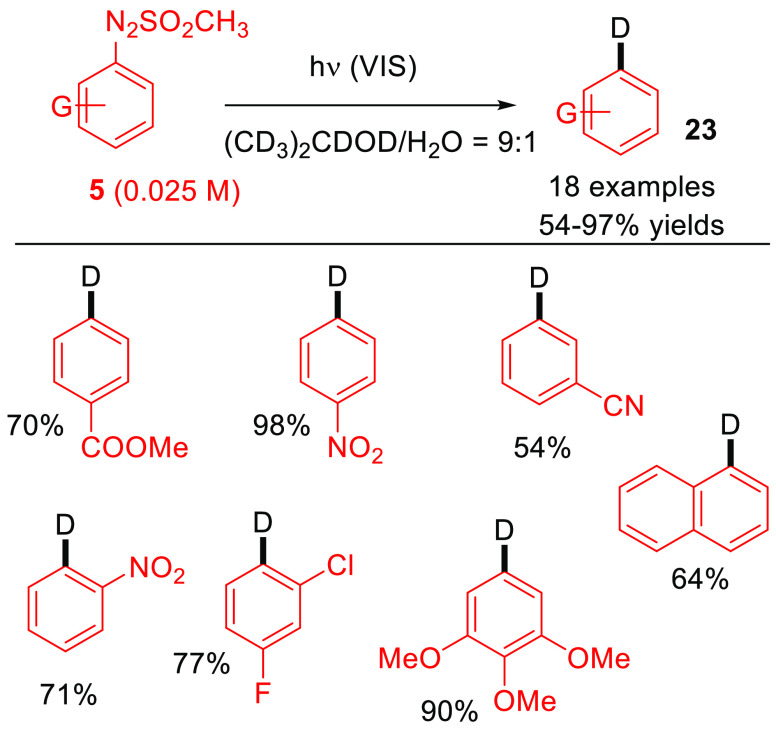
Visible-Light-Driven Deutero Deamination of Arylazo Sulfones. General Procedure and Selected Examples

Deuterated compounds find application in the field of mass and NMR spectroscopy, and methods for the construction of aryl–D bonds were variously developed in the past decade, some of them exploiting photoredox catalysis.^51^ A set of monodeuterated aromatics **23** was instead obtained via a catalyst-free visible-light-driven deutero deamination of arylazo sulfones in the presence of either aqueous isopropanol-*d*_7_ or tetrahydrofuran-*d*_8_ as deuterium sources. Notably, the presence of a significant amount of water did not appreciably affect the deuteration yield (Scheme 14).^52^

## The Dyedauxiliary −N_2_SO_2_R: Leaving Group or Reactant?

As stated in Scheme 3, different reactive intermediates may be generated during the irradiation of **5**, most of them playing a key role in the processes described above. In all cases, the leaving group D is released and is lost in the process. However, in some cases, part of the dyedauxiliary group is incorporated in the final product, thus further highlighting the versatility of arylazo sulfones chemistry. As an example, trapping of the generated diazenyl radical by the π-bond system can occur before N_2_ loss. This behavior was exploited for the diazenylation of enol silyl ethers to form a set of aza derivatives that exhibit bioactive properties and that found application in the synthesis of *N*-containing heterocycles.^53^

**Scheme 15:**
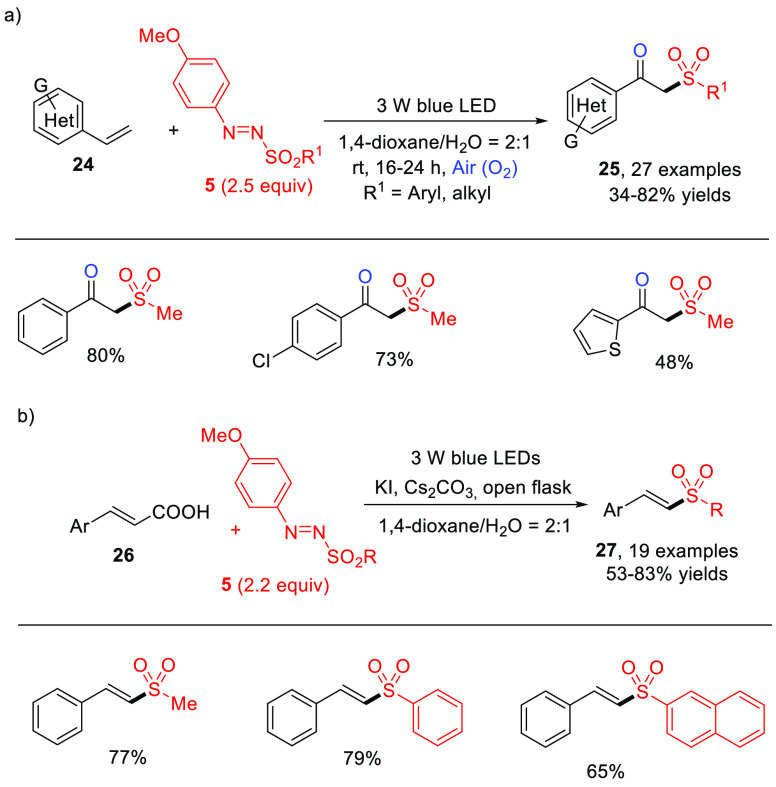
Photocatalyst-Free Visible-Light-Induced Synthesis of (a) β-Oxo Sulfones **25** via Oxysulfonylation of Aromatic Alkenes and (b) of Vinyl Sulfones **27** from Cinnamic Acids and Arylazo Sulfones (General Procedure and Selected Examples)

The same methanesulfonyl radical generated via visible light photolysis of **5** has been used for synthetic purposes. In fact, in 2019, Wei and co-workers reported the visible-light-induced oxysulfonylation of alkenes in the presence of arylazo sulfones and oxygen operated by the sulfonyl radical produced. In the protocol, a series of functionalized β-oxo sulfones **25** were synthesized at room temperature via oxidative difunctionalization of styrenes **24** (Scheme 15a).^54^ Later, the same group employed an analogous approach to prepare α-sulfonyl ketones from alkynes, again making use of 4-methoxyphenylazo sulfones as the sulfonylating agents.^55^

In 2020, Yadav developed a way to access (*E*)-vinyl sulfones **27** in moderate to high yields via sulfonylation/decarboxylation of cinnamic acids (**26**, Scheme 15b) upon blue LED irradiation.^56^

## Application of Arylazo Sulfones in Material Sciences

**Scheme 16:**
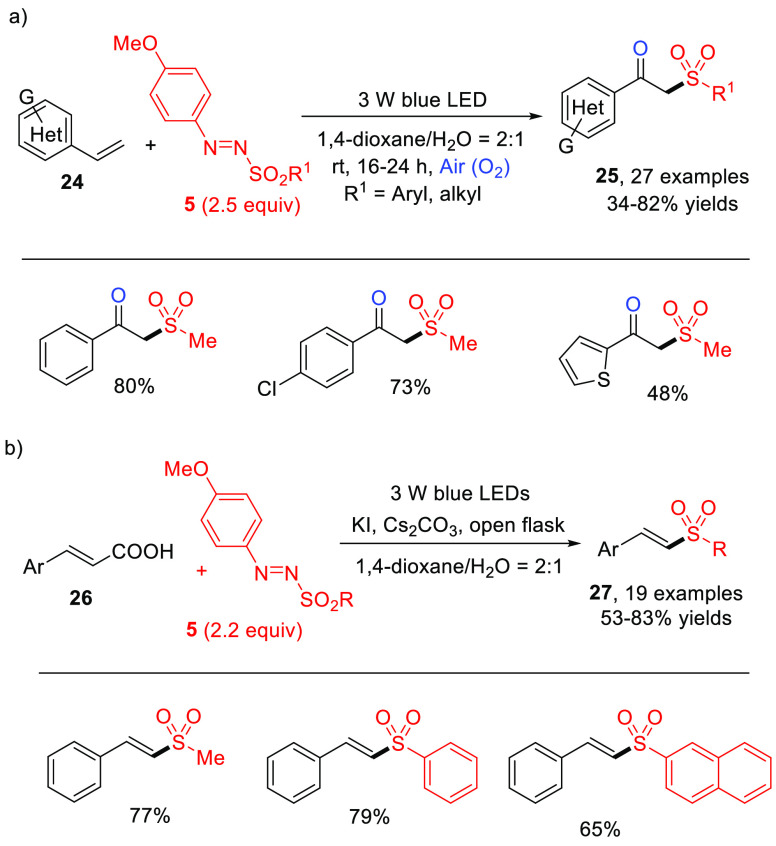
Visible-Light Photografting of Aryl and Methanesulfonyl Groups on a Gold Surface Adapted from ref (60). Copyright 2020 American Chemical Society.

The use of arylazo sulfones as thermal^57,58^ and (rarely) photochemical^59^ initiators in the polymerization of methacrylate esters has been sparsely reported. The simultaneous photografting of both differently substituted aryl and methanesulfonyl groups on a gold surface was achieved via the N–S photoinduced cleavage of arylazo sulfones **5** and trapping of the generated aryl/methanesulfonyl radical pair (Scheme 16).^60^ The developed approach simply involves visible light as the only promoting agent of **5** and avoids the use of electrografting or photoredox-catalyzed processes commonly employed for the surface functionalization via onium salts.

**Figure 4:**
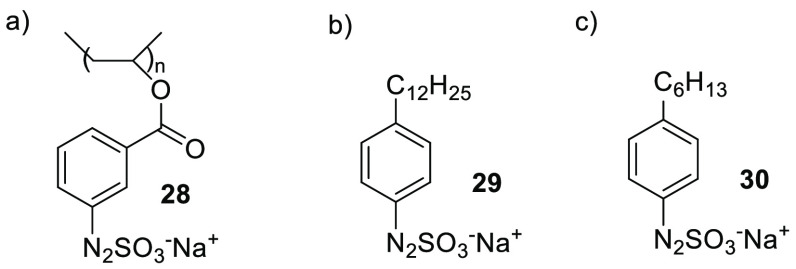
Photoreactivearylazo Sulfonates **28**–**30**.

In this context, however, more attention has been offered to the related arylazo sulfonates, water-soluble compounds that could be easily prepared by treating the corresponding arenediazonium salt with aqueous Na_2_SO_3_ in the presence of a base (e.g., Na_2_CO_3_). The azosulfonate chromophore can be incorporated as a side group into a polymer, and the resulting photoresin is exploited in offset printing techniques and photolithography.^61^ Recently, a set of water-based azosulfonate-doped poly(vinyl alcohols) (**28**, Figure 4a) was prepared and tested as a highly thermally stable photoresist material.^62^ Dunkin et al. exploited the reactivity of arylazo sulfonates to develop a new class of visible-light photoactive surfactants (**29**, Figure 4b)^63^ that were later employed as photolabile emulsifiers in the polymerization of methylmethacrylate.^64^

**Figure 5:**
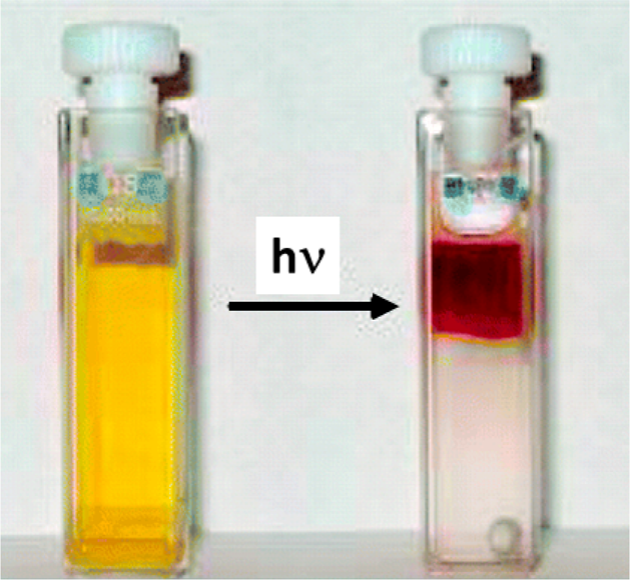
Phase separation transition after UV irradiation of aqueous 85 mM **30**/ hexaethylene glycol mixtures in 0.5 M NaCl. Adapted from ref (66). Copyright 2005 American Chemical Society.

Sodium 4-hexylphenylazosulfonate **30** (Figure 4c) was used as photolabile surfactant in photoresposive emulsions.^65^ Thus, aqueous systems, containing nanoscopic micellar aggregates obtained by the simultaneous presence of photolabile **30** and the inert nonionic hexaethylene glycol as the surfactants, undergo macroscopic phase separation via light-driven photolysis of **30** (see Figure 5).^66^

## Outlook and Conclusions

Visible-light irradiation is now considered as a standard condition in organic synthesis, but in most cases its use is strictly related to the presence of a photocatalyst.^5,31b^ As a matter of fact, this approach stated the role of photons to that of an energy source alternative to conventional heating, while the reaction course (occurring via energy, electron or atom transfer)^5^ depends on the reactivity of the excited state of the photocatalyst. This is not necessarily a limitation, since an impressive versatility and efficiency has been demonstrated for most of these protocols, but, however, the potential of photons as green reactants, able to directly cleave/form a chemical bond, is unexpressed.

In view of these premises, the generation of a wide range of reactive intermediates upon direct visible-light irradiation of the reactant would be, in the opinion of the authors, a further, evolutionary leap for organic photochemistry that will underline the role of light as a green reactant in synthesis. In this context, as recently demonstrated, the *dyedauxiliary group strategy* represents a promising approach to make a wide range of highly reactive intermediates in modern organic synthesis easily accessible.
